# Healthcare and End-of-Life Needs of Lesbian, Gay, Bisexual, and Transgender (LGBT) Older Adults: A Scoping Review

**DOI:** 10.3390/geriatrics2010013

**Published:** 2017-03-16

**Authors:** Arne Stinchcombe, Jeffrey Smallbone, Kimberley Wilson, Katherine Kortes-Miller

**Affiliations:** 1 School of Psychology, University of Ottawa, Ottawa, ON, K1N 6N5, Canada; 2 Department of Health Sciences, Lakehead University, Thunder Bay, ON, P7B 5E1, Canada; 3 Department of Family Relations and Applied Nutrition, University of Guelph, Guelph, ON, N1G 2W1, Canada; jsmallbo@mail.uoguelph.ca (J.S.); kim.wilson@uoguelph.ca (K.W.); 4 School of Social Work, Lakehead University, Thunder Bay, ON, P7B 5E1, Canada; kkortesm@lakeheadu.ca; 5 Centre for Education and Research on Aging and Health (CERAH), Lakehead University, Thunder Bay, ON, P7B 5E1, Canada

**Keywords:** end-of-life, healthcare, LGBT, lesbian, gay, bisexual, transgender, aging, older adults

## Abstract

Lesbian, gay, bisexual, and transgender (LGBT) older adults face a number of challenges with respect to access to healthcare especially towards end-of-life. Through a systematic search and scoping review of the literature, we sought to answer two related research questions. In particular, the purpose of this scoping review was to determine the healthcare needs of LGBT older adults nearing end-of-life as well as the factors that contribute to a good death experience among older adults who identify as LGBT. A systematic search of electronic databases for articles published between 2005 and 2016 as well as screening for relevance resulted in 25 results. The data were charted and grouped according to the themes of: social support and chosen family, intimacy, health status, fear of discrimination and lack of trust, lack of knowledge and preparedness, and cultural competence in the healthcare system. The results suggest a role for health and social service workers in contributing to a positive care experience for LGBT older adults by becoming knowledgeable about the unique needs of this population and being unassuming and accepting of individuals’ sexuality. Many of the articles reviewed collected data outside of Canada, limiting generalizability and highlighting a need for Canadian data on LGBT aging and end-of-life.

## 1. Introduction

Researchers have reported disparities in health between older LGBT individuals and their non-LGBT peers, including less access to healthcare and the negative effects of stigmatization [1]. In a sizeable US-based study, LGBT individuals reported concerns regarding social isolation, finances, and access to health services. Limited Canadian data suggest that older LGBT individuals are more likely to live alone and be socially isolated, and have a strong desire to avoid long-term care facilities (i.e., institutionalized care) [2]. 

LGBT individuals often experience stigma and discrimination, which can have a negative impact on their health and even their life expectancy. For example, Hatzenbuehler and colleagues showed that sexual minorities living in neighbourhoods with high levels of anti-gay prejudice experienced shorter life expectancies of 12 years, compared to peers living in low-prejudice communities [3]. As well as experiencing health disparities, stigma, and discrimination, older LGBT individuals are less likely than their majority peers to have spouses and kin to serve as caregivers, provide guidance in navigating the healthcare system, and support them in making decisions in the last stages of life [4]. Support networks of LGBT persons are often made up of friends and neighbours (i.e., chosen family), as opposed to spouses and kin, which can further complicate decision-making later in life, especially if powers of attorney have not been designated. 

To date, available data clearly outline that LGBT older adults and their caregivers face unique challenges with respect to health care and decision-making in the later stages of life. We sought to better understand the experiences of older LGBT individuals within the healthcare system as well as highlight considerations unique to this population regarding care in the last stages of life. Given the changing policy and practice landscapes around death and dying in Canada, this review emphasizes the transferability of existing research to the Canadian context. Through a scoping review of the literature, our goal was to answer two related research questions. In particular, the purpose of this scoping review was to determine the healthcare needs of LGBT older adults nearing end-of-life as well as the factors that contribute to a good death experience among older adults who identify as LGBT.

## 2. Method

Scoping reviews are most appropriate when there is minimal literature on a particular topic, when methodological approaches on the topic of interest are diverse, and/or when the research question is broad [5]. Scoping reviews aim to map important concepts in a research domain and serve to examine the breadth and depth of knowledge, summarize research findings, and identify knowledge gaps [5]. The current literature on LGBT aging and end-of-life is varied in terms of methodological approach, thus aligning with the scoping review methodology. A knowledge synthesis was executed using a scoping review approach wherein the goal was to map the literature on a particular topic, identify key concepts, and explore data sources. The researchers drew from the approach proposed by Levac, Colquhoun and O’Brien [6] to undertake the scoping review. Unlike a systematic review, a scoping review does not assess the quality of the studies included in the review [6]. 

Aligning with key legislative decisions in Canada (i.e., Civil Marriage Act in 2005), electronic searches included articles published between 2005 and 2016 (i.e., 11 years). The following databases were searched: AARP Ageline, Women’s Studies International, Web of Science Core Collection (includes Social Sciences Citation Index), CINAHL, MEDLINE, PsycINFO, PsycARTICLES, Cochrane Database of Systematic Reviews, and Nursing & Allied Health Database. Additional resources were obtained by examining the reference lists of retrieved articles and by searching Google. The search contained key words denoting the last stages of life (e.g., end-of-life, palliate*, dying, etc.), older adulthood (e.g., senior, aging, elder, etc.), and LGBT (e.g., lesbian, gay, bisexual, etc.). The search was initially developed in Medline then translated based on the search parameters for each of the databases. 

**Figure 1:**
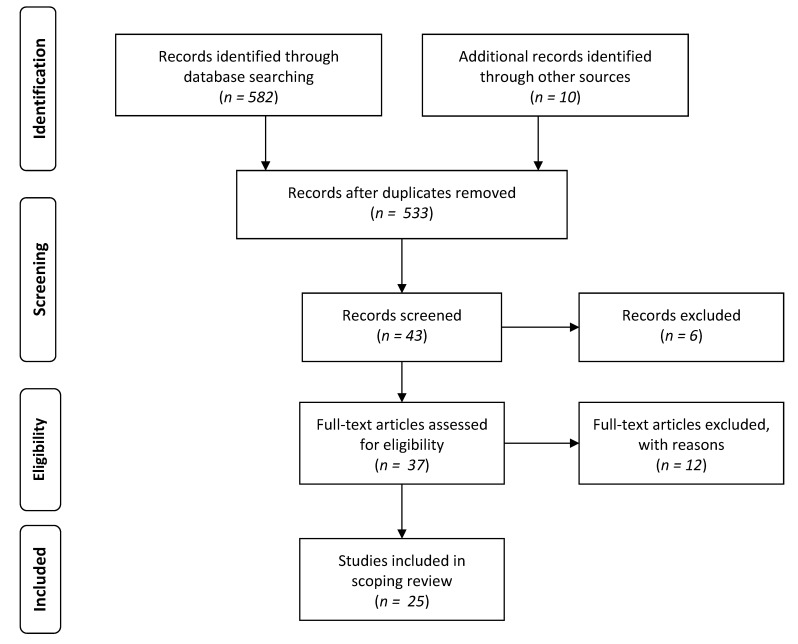
Flow diagram for scoping review showing literature search and selection.

All peer-reviewed articles that pertained to aging, end-of-life, and LGBT individuals were retained for review. A flow chart detailing the article selection process can be found in Figure 1. 

Following the relevancy review, two of the authors independently reviewed each of the articles, identifying key concepts. Through a collaborative process, the two researchers met to discuss the development and naming of the themes. Differences between the researchers were resolved through discussion and consensus. 

## 3. Results

In total, 25 articles were selected for inclusion in the review. As part of the scoping review process, each of the articles was charted in MS Excel where the article authors, year of publication, sample information, and research design were detailed (see Appendix A). A brief summary of each article, including key findings, was also generated by the researchers and assembled as an annotated bibliography, which was subsequently independently reviewed by two of the researchers. 

The included articles varied in terms of methodology and country of origin; the majority were observational in nature and originated from the United States and Australia. Several themes emerged from the review of included literature that were identified as the following:

1. social support and chosen family;
2. intimacy;
3. health status;
4. fear of discrimination and lack of trust;
5. lack of knowledge and preparedness; and
6. cultural competence in the healthcare system.

These themes were added to the charting of data and into the table (see Appendix A). We review each of these principal themes from the literature below. 

### 3.1. Social Support and Chosen Family

The existing literature emphasizes the importance of family, friends, and communities for the well-being of older LGBT individuals. Research also highlights a role for *chosen family* (also referred to as *families of choice* and *lavender families* [7]) not only for providing social support and caregiving but also for end-of-life decision-making. Chosen family refers to friends and community members who provide support, companionship, and love in preference to biological relatives [8]. Evidence suggests that it is important to include “loved ones” or “family” as defined by the patient in the decision-making process for palliative and end-of-life care [9]. For example, findings from the MetLife survey, a cross-sectional study of 1000 US-based LGBT baby boomers, highlighted that respondents were highly involved in the provision of informal care for their families and that non-familial social networks play an important role as a source of social support [10]. Similarly, a small qualitative study of 15 older gay and lesbian adults in the United Kingdom described the complex nature of the familial relationships of participants’ including a mix of biological and non-biological members [11]. The article suggests that LGBT individuals may prefer a close friend to have legal rights regarding their end-of-life care and decisions rather than a biological relative. The authors of the latter study commented on the importance of social support for continued well-being amongst this population. 

### 3.2. Intimacy

Older adults continue to have sexual and intimacy needs in the presence of declining health and end-of-life care; this is true for heterosexual individuals as well as for queer-identified individuals. However, the needs of LGBT older adults within formal care settings may be overlooked because of heteronormative and/or cisnormative assumptions from health and social care workers, as well as fear of disclosure on the part of patients and families. 

In a review of the literature on the palliative and end-of-life care needs of LGBT individuals, Griebling points out that sexuality is a core component of our experience as humans and deserves to be included in palliative care settings [9]. Declining health and the presence of terminal illness that accompanies end-of-life often has negative impacts on sexual functioning. Griebling indicates that existing literature shows that simple intimacy, physical closeness, and emotional connectedness often become more important than sexual intercourse among those in the last stages of life. The author draws many parallels between the needs of heterosexual and LGBT individuals close to end-of-life. An important distinction, however, is the experience of LGBT individuals with respect to the negative attitudes held by others. This may reduce an individual’s willingness to communicate with health and social care professionals about their sexual and intimacy needs within care settings including hospice. 

### 3.3. Health Status 

The existing research emphasizes the intersections between sexual minority statuses, physical and mental health, and the receipt of end-of-life care. In particular, declines in physical health typically accompany the last stages of life and there are often mental health consequences, such as depression [9]. In general, LGBT individuals are at a greater risk of negative physical and mental health outcomes relative to the general population and these needs should be considered in the last stages of life [12]. Indeed, research suggests that these negative health outcomes among LGBT older adults may be partly mitigated by the presence of social support [13]. Thus, support from others is an important consideration for LGBT older adults as well as when entering into the last stages of life. 

Aging is associated with an increased risk of cognitive impairment, such as dementia, that is especially problematic for LGBT individuals. In particular, LGBT individuals with dementia may be triply marginalized within care settings due to the intersection of their age, dementia/cognitive status, and sexual identity, leading to poor quality care [14]. In a review paper by McGovern, the author identifies a lack of research on older LGBT individuals with dementia and, within the research that does exist, a particular invisibility of older transgender individuals [14]. The author argues that more research in field is vital to providing appropriate health and social care to this community of older adults. 

### 3.4. Fear of Discrimination and Lack of Trust

A theme that emerged in the literature is fear regarding end-of-life including chronic disease and disability as well as fear of discrimination within the medical and legal systems. More specifically, in a large study of 1963 trans-identified adults that focused on later life and end-of-life preparations, Witten observed that participants expressed fear about the potential late-life events and end-of-life needs [15]. These included concerns about legal requirements and care needs. Other research highlights the fears of discrimination due to sexual orientation within long-term care settings as well as fear over discriminatory care [8,12,16]. 

Older LGBT individuals often have concerns about the medical system and distrust of health and social care providers. They have expressed anxiety about the possibility of receiving care from a provider who is homophobic [7]. In a small study of 19 gay and lesbian adults over the age of 50, many participants voiced anxiety over potential social isolation and rejection during end-of-life care due to their sexuality [17]. Approximately half of the sample was against the idea of assisted living while over 80% of the sample was against the idea of living in long-term care. These concerns stem from fear that staff would not be knowledgeable about lesbian and gay-specific issues. 

However, the notion that all members in the LGBT community are distrustful of the healthcare system is not consistent across the literature. In fact, June and colleagues [18] reported no differences between heterosexual women and lesbian woman in the state of Colorado (USA) in terms of distrust of the healthcare system. This latter finding may result from regional differences in attitudes towards healthcare and end-of-life as well as jurisdictional differences in the availability of LGBT-friendly health services. 

With respect to the legal system, LGBT individuals report distrust and fear that their advance directives will not be respected due to their sexual minority status [19]. This fear may originate from a lifetime of discrimination and/or experiences interfacing with the medico-legal system. Data from the US indicate that older lesbians report having struggled with policies that ignore their lifetime romantic partnerships and thereby deny them end-of-life decision-making and access to death benefits [19]. 

### 3.5. Lack of Knowledge and Preparedness

Overall, the literature indicates that LGBT individuals lack knowledge about the legal provisions that can help ensure their end-of-life decisions are respected. As such, few individuals have appropriately planned for end-of-life [10]. A study that originated from Australia suggests that while a majority of LGBT individuals were aware of end-of-life care planning options, few of them had actually used these options [20]. When participants were presented with a fictitious scenario, only 27% of the sample was able to correctly identify who had the legal right to make treatment decisions for an unconscious person following a car crash. The findings also indicated that while 76% of respondents were comfortable with their healthcare provider raising end-of-life issues with them, transgender and closeted respondents were less comfortable having these conversations with their healthcare provider. Another study conducted by the same authors suggests that while advance care planning options have the potential to promote the rights of LGBT people at the end of life (insofar as it allows them to choose precisely who they would like to make decisions on their behalf), their levels of uptake approximate those of the general population [21]. 

A survey of 1963 transgender adults found that respondents had major concerns about end-of-life that were integrated with concerns about chronic illness and disability [15]. In general, the findings indicated that respondents were not prepared for major legal issues and events that take place in the last stages of life and reported fears about the future. In a follow-up study using the same database but focusing on transgender-identified lesbians, Witten similarly found that respondents were poorly prepared for end-of-life [22]. The author suggests that lack of end-of-life legal protections and documentation serve as barriers to this particular population and more measures should be implemented in order to remedy this issue.

Lack of education regarding the legal provisions for end-of-life care among health and social care workers as well as among LGBT individuals has been cited as an obstacle to improving care [23]. Thus, as echoed elsewhere in the literature, educating LGBT individuals as well as health and social care providers on existing legal provisions is necessary to prevent discrimination in the last stages and ensure quality of healthcare as well as the uptake of planning tools [24,25]. Taken together, these findings suggest that while there is broad awareness of end-of-life care planning options, LGBT individuals would benefit from more information through education efforts.

### 3.6. Cultural Competence in the Healthcare System

Many LGBT individuals are presented with heteronormative and cisnormative assumptions when interfacing with healthcare and social service providers [9,24,26] and these assumptions in turn may contribute to a lack of trust towards the healthcare system. Negative societal attitudes, discrimination, homophobia, and transphobia shape end-of-life experiences for LGBT individuals and care providers need to consider these when offering care [7]. 

Recent efforts have been made to develop educational materials for healthcare workers to address the needs of LGBT individuals [9]. However, as Aldredge and Conlon note, care providers often have a heteronormative outlook and lack of professional education on LGBT for palliative and end-of-life care [27]. These observations suggest that while there is effort to educate healthcare professionals, there may be barriers to accessing and implementing culturally competent care approaches that would facilitate inclusion of LGBT individuals in the last stages of life.

Communication between patients and healthcare providers remains a barrier in the provision of appropriate care for LGBT patients. A recent review of the recommendations for techniques to improve communication among care providers and recipients who identify as LGBT conducted by Lawton and colleagues suggests that clinicians can reduce barriers by having an open and affirming approach to interactions with patients and their families [28]. They suggest that clinicians should use inclusive language when interacting with patients. Additionally, clinicians should work to assure confidentiality, privacy, and professionalism in their interactions with patients. 

Much of the research has focused on the role of health and social care workers in reducing barriers for LGBT older adults. Arthur completed a review of the literature which sought to ascertain whether current social, legal, and medical care is meeting the unique needs of the LGBT older adults [16]. The author suggests that health and social care professionals are not well prepared to work with LGBT older adults and that LGBT older adults have expressed concerns and fear over discriminatory care. Arthur concludes that there is a widespread lack of recognition of the unique needs faced by LGBT older adults during end-of-life and palliative care. 

Through a review of four case studies, Duffy and Healy explain that while successive generations of LGBT cohorts are being perceived in a more positive light, the current cohort of older-old (i.e., 85 years and older) LGBT individuals face the greatest number of barriers and that service providers who interact with this demographic should be particularly sensitive to their needs [29]. Other authors indicate that palliative care workers should focus on dignity, equality, and respect in order to improve the quality of service provided to LGBT individuals [7]. Kimmell argues that gerontologists can improve care to this demographic by recognizing how sexual orientation reflects an individual’s experiences and that it is imperative that those working with older adults be unassuming and accepting of an individual’s sexuality [12]. 

Price suggests that service providers be educated in the issues specifically faced by these individuals such as social isolation from their biological families due to their sexuality [1]. It is also suggested that health care providers should be open to the possibility that caregivers of LGBT individuals may be members of the LGBT community themselves, and should also respect an individual’s degree or lack of openness in sharing information about their sexuality. Price states that it is necessary to acknowledge the biases and barriers faced by lesbian and individuals in terms of receiving adequate end-of-life care in order to improve end-of-life care. Price suggests that training healthcare providers to be aware of and knowledgeable about LGBT specific issues could improve the care that they receive.

Existing research suggests that efforts to train health and social care workers can effectively increase knowledge and awareness about issues facing older LGBT adults. 

Porter and Krinsky, for example, held four training events in Massachusetts to determine the efficacy of LGBT aging competency training for service providers who work with older adults [30]. The results indicated significant improvements in the areas of knowledge, behavioural intentions, and attitudes towards the LGBT community following training. Specifically, results showed improved knowledge of policies (e.g., legal, financial, and health) and resources (e.g., informational, support, etc.) relating to LGBT individuals. Post-test results also indicated that participants felt more comfortable providing care to lesbian, gay and bisexual (LGB) individuals following training; however, they also felt less comfortable providing care to transgender (T) individuals. The authors attribute this discrepancy to the possibility that after learning of the unique needs of this group, the participants did not feel as confident in their ability. These findings show that LGBT-aging training is beneficial for service providers who are likely working with LGBT clients.

## 4. Discussion

Through this scoping review, the available literature on LGBT aging and the last stages of life bring to light a number of important findings. First, it is clear from the available literature that LGBT older adults are at greater risk of physical and mental health conditions in comparison to the general population but that social support may mitigate some of these negative outcomes. In a similar vein, the literature emphasizes that LGBT older adults include friends in their chosen families and have a desire for them to play an active role in their care and end-of-life decision-making. 

In addition, within formal care settings, LGBT older adults fear discrimination and do not trust that the medical or legal systems will carry out their end-of-life wishes. Part of this lack of trust may derive from a lack of knowledge about end-of-life care planning options combined with lack of preparedness. Together, these factors place LGBT older adults at increased risk for lower quality of care in the last stages of life. 

Older LGBT individuals face discrimination within the medical system and cognitively impaired LGBT individuals may at greater risk of experiencing stigma and discrimination due to the intersection of chronological age, sexual identity, and cognitive health. LGBT older adults have intimacy and sexual needs in the last stages of life but these needs often go unmet because of negative attitudes held by health and social workers. However, despite discrimination faced by LGBT older adults within the healthcare system, it is clear that health and social service workers can play a critical role in contributing to a positive care experience for LGBT older adults and their families by becoming knowledgeable about the unique needs of this population and being unassuming and accepting of individuals’ sexuality. 

The results of this scoping review are useful in mapping the existing literature pertaining to LGBT aging and the last stages of life. The results are diverse in their methodologies yet many came to similar conclusions with respect the concerns and experiences of LGBT older adults and end-of-life care. Overall, the literature demonstrates that LGBT persons entering the last stages of life face multiple challenges receiving quality care. These challenges include lack of confidence that the health care system will address their needs, fear of stigma and discrimination, lack of knowledge about legal tools (e.g., advance care planning), and inadequate education for service providers, which could serve to mitigate discriminatory effects on LGBT individuals. We also sought to explore factors that contribute to a good death experience. While this was not an explicit component of the included research literature, taken together the emergent themes presented here illustrate elements that facilitate quality of living until the end of life.

There are, however, limitations to the available literature. In particular, there were few comparative studies that contrast the experiences of LGBT participants with their heterosexual/cisgendered peers. It is possible that some of themes from this body of this literature may also apply to majority populations as well. Another important limitation to the above-cited research is that much of the data were collected in jurisdictions with differing historical and medico-legal contexts, potentially limiting the transferability of findings. For example, in reference to Canada, where the authors of this paper reside, the few articles that collected data from Canadians included Brotman and colleagues and Witten where 6% of the sample resided in Canada [8,22]. Indeed, some of the discrepancies observed in the literature may reflect geographic, cultural, or jurisdictional differences in the experiences of older LGBT individuals and their levels of comfort with the medico-legal systems. Additionally, this observation about limitations in the available literature is not unique to jurisdictional issues, as numerous articles included in this scoping review also call for more research in this area more generally (e.g., [14]). Transgender individuals have unique and specific physical health needs (e.g., hormone therapy). In many of the studies reviewed, transgender and bisexual individuals were not represented to the same extent as gay and lesbian individuals. Transgender older adults in particular represent a population for which knowledge about the aging experience and appropriate end-of-life care is especially lacking. Researchers, policy makers, and practitioners should exercise caution so as not to interpret findings from research studies focused on lesbian and gay individuals as representative of the experiences of bisexual or transgender individuals. 

## 5. Conclusions

From a policy and practice standpoint, the available literature highlights the importance of promoting inclusion at end-of-life and addressing the specific needs of LGBT individuals. Healthcare providers should work towards creating safe spaces for and building rapport with LGBT patients, to minimize fear of discrimination. Similarly, educational resources should be made available to healthcare providers, enabling them to provide culturally competent end-of-life care. These efforts may help to reduce fears of mistreatment at end of life and reduce the occurrence of actual experiences of discrimination. 

To some extent, our ability to draw inferences about the experience of LGBT older adults across jurisdictions is limited given the current state of the literature. Continued research in this area is particularly timely given that several countries are experiencing a demographic shift and a movement towards inclusivity. This shift is noted through reports on LGBT aging and end of life released in the United Kingdom [31] and Australia [32]. Taking a life course perspective necessitates an understanding of the personal histories and historical context in which LGBT older adults are situated [33]. In Canada, for example, major historical milestones aiming to guarantee equal civil liberties members of the LGBT community include the decriminalization of homosexuality in 1969 as well as the Civil Marriage Act in 2005, which allowed same-sex couples access to marriage. It is similarly important to note that the rights of trans individuals in Canada continue to lag behind those of other members of the LGBT community and vary considerably depending on jurisdiction [34]. Undeniably, the experience of Canadian older LGBT individuals is unique relative to LGBT individuals residing in countries around the world. Thus, there is a gap in the literature on LGBT aging in Canada and, more precisely, the last stages of life. In order to better understand the experiences of LGBT older adults with respect to the last stages of life, more jurisdiction-specific research is needed as is a focus on the needs of the transgender community at the end of their lives. 

## Appendix A.

**Table A1:** Charted articles included in the scoping review.

No.	Author Information	Sample Information	Research Design	Relevant Themes
1	Brotman et al. 2007 [8]	Age: Range = 33–72; mean/median = n/a ^1a^ Gender: M and F Sample Size: 17 Location: Quebec, British Columbia, Halifax	Method: QualitativeDesign: Cross-sectional Collection: Interviews Analysis: Qualitative analysis	Social support and chosen family, fear of discrimination and lack of trust,
2	Griebling 2016 [9]	n/a	Design: Synthesis paper	Social support and chosen family, intimacy, health status, cultural competence in the healthcare system
3	Metlife Mature Market Institute et al. 2010 [10]	Age: Range = 40–61; mean/median = n/a Gender: M and F including ftm and mtf transgender individuals Sample Size: 1000 Location: United States, Canada, Thailand, Australia, Sweden, Mexico	Method: Mixed-method Design: Cross-sectional Collection: Interviews and Questionnaires Analysis: descriptive statistics, confidence intervals,	Social support and chosen family, lack of knowledge and preparedness
4	Almack et al. 2010 [11]	Age: Range = 55–84; mean/median = n/a ^2a^ Gender: M and F Sample Size: 15 Location: United Kingdom	Method: Qualitative Design: Cross-sectional Collection: Focus groups Analysis: Qualitative analysis	Social support and chosen family
5	Harding et al. 2012 [24]	n/a	Design: Synthesis paper	Lack of knowledge and preparedness, cultural competence in the healthcare system
6	Corbett 2007 [26]	Age: Range = 23–65; mean/median = n/a Gender: M and F Sample Size: 27 Location: Uppsala, Sweden	Method: Qualitative Design: Cross-sectional Collection: Interviews Analysis: Qualitative analysis	Cultural competence in the healthcare system
7	Rawlings 2012 [7]	n/a	Design: Synthesis paper	Social support and chosen family, fear of discrimination and lack of trust, Cultural competence in the healthcare system
8	Aldredge et al. 2012 [27]	Age: Range = n/a ^3a^; mean/median = n/a ^3a^ Gender: M and F Sample Size: 6 Location: United States	Method: Qualitative Design: Cross-sectional Collection: Reflective works Analysis: Qualitative analysis	Cultural competence in the healthcare system
9	Lawton et al. 2014 [28]	n/a	Design: Synthesis paper	Cultural competence in the healthcare system
10	Arthur 2015 [16]	n/a	Design: Synthesis paper	Cultural competence in the healthcare system, fear of discrimination and lack of trust
11	Duffy et al. 2014 [29]	Age: Range = early 60s–78; mean/median = n/a Gender: M and F Sample Size: 4 Location: Sydney, Australia	Method: Qualitative Design: Case-study Collection: Interview Analysis: Qualitative analysis	Cultural competence in the healthcare system
12	Kimmel 2014 [12]	n/a	Design: Synthesis paper	Health status, fear of discrimination and lack of trust, cultural competence in the healthcare system
13	Porter et al. 2014 [30]	Age: Range = n/a ^4a^; mean/median = n/a ^4a^ Gender: M and F Sample Size: 76 Location: United States	Method: Quantitative Design: Case series Collection: Questionnaires Analysis: Descriptive statistics, t-tests, chi-square analyses, ANOVAs,	Cultural competence in the healthcare system
14	Price 2005 [1]	n/a	Design: Synthesis paper	Cultural competence in the healthcare system, fear of discrimination and lack of trust
15	Hughes et al. 2014 [20]	Age: Range = <40 to 70+; mean/median = n/a Gender: M, F, Transgender, Intersex Sample Size: 305 Location: New South Wales, Australia	Method: Mixed-method Design: Cross-sectional Collection: Questionnaires Analysis: Qualitative analysis, descriptive statistics, chi-square	Lack of knowledge and preparedness
16	Hughes et al. 2015 [21]	Age: Range = <40 to 70+; mean/median = n/a Gender: M, F, Transgender, Intersex Sample Size: 305 Location: New South Wales, Australia	Method: Mixed-method Design: Cross-sectional Collection: Qualitative analysis, descriptive statistics, chi-square analysis, bivariate analysis	Lack of knowledge and preparedness
17	Cartwright et al. 2012 [23]	Age: Range = n/a; mean/median = n/a ^4a^ Gender: M and F Sample Size: 25 Location: New South Wales, Australia	Method: Qualitative Design: Cross-sectional Collection: Interviews Analysis: Qualitative analysis	Lack of knowledge and preparedness,
18	Powell et al. 2012 [25]	n/a	Design: Synthesis paper	Lack of knowledge and preparedness
19	Witten et al. 2014 [15]	Age: Range = 18 to 51 and over ; mean/median = n/a Gender: mtf and ftm transgender Sample Size: 1,963 Location: United States, Canada, Thailand, Australia, Sweden, Mexico	Method: Mixed Method Design: Cross-sectional Collection: Questionnaires, Interviews Analysis: Means, descriptive statistics, confidence intervals, chi-square analysis, t-tests, qualitative analysis,	Fear of discrimination and lack of trust, lack of knowledge and preparedness
20	Witten et al. 2015 [22]	Age: Range = 18 to 51 and over; mean/median = n/a Gender: transgender ^5a^ Sample Size: 276 Location: United States, Canada, Thailand, Australia, Sweden, Mexico	Method: Mixed Method Design: Cross-sectional Collection: Questionnaires, Interviews Analysis: Means, descriptive statistics, confidence intervals, chi-square, t-test, qualitative analysis,	Lack of knowledge and preparedness, Fear of discrimination and lack of trust
21	Hash et al. 2007 [17]	Age: Range = 50–77; mean = 60 Gender: M and F Sample Size: 19 Location: United States	Method: Qualitative Design: Cross-sectional Collection: Interviews Analysis: Qualitative analysis, descriptive statistics	Fear of discrimination and lack of trust
22	June et al. 2012 [18]	Age: Range = 60–81; mean = 64.8 Gender: F Sample Size: 30 Location: Colorado, United States	Method: Quantitative Design: Cross-sectional Collection: Questionnaire Analysis: t-tests, chi-square, analysis of covariance (ANCOVA),	Fear of discrimination and lack of trust
23	Averett et al. 2011 [19]	Age: Range= 51–86; median = 62 Gender: F Sample Size: 456 Location: United States	Method: Mixed-method Design: Cross-sectional Collection: Questionnaire Analysis: Descriptive statistics, means	Fear of discrimination and lack of trust
24	Masini et al. 2008 [13]	Age: Range = 50–79; mean = 57 Gender: M and F Sample Size: 220 Location: United States	Method: Quantitative Design: Cross-sectional Collection: Questionnaire Analysis: Descriptive statistics, multiple regression, chi-square analysis, frequencies	Health status
25	McGovern 2014 [14]	n/a	Design: Synthesis paper	Health status

^1a^ Mean age of partners = 63; mean age of adult children = 43; ^2a^ 5 people in the study did not disclose their age; 1 support worker under the age of 45 took part in the study; ^3a^ 6 participants were healthcare workers recounting stories of elderly LGBT patients, but did not provide exact ages; ^4a^ Participants were the service providers; ^5a^ Gender identity was self-reported and the study included those who identified as “trans-lesbians”.
